# Facilitators and barriers of patient-centered care at the organizational-level: a study of three district hospitals in the central region of Ghana

**DOI:** 10.1186/s12913-019-4748-z

**Published:** 2019-11-27

**Authors:** Jacqueline Nkrumah, Gordon Abekah-Nkrumah

**Affiliations:** 1 Department of Health Administration and Education, Faculty of Science Education, University of Education, Winneba, P.O Box 25, Winneba, Central Region, West African Ghana; 2 Department of Public Administration and Health Services Management, University of Ghana Business School, P. O. Box, 75, Accra-Legon, Greater Accra Region, West Africa Ghana

**Keywords:** Patient-centered care, Organizational-level factors, Patient-centered care facilitators and barriers

## Abstract

**Background:**

Improving patient experience of care has gained enormous attention from policy makers and providers of healthcare services in Ghana. In spite of the supposed support for patient-centered care as the means for improving patient experience of care, scientific evidence point to poor patient experience of care in Ghana. Moreover, there seem to be little evidence on organizational-level factors that facilitate or hamper patient-centered care. In this study we assess organizational-level factors that facilitate or impede patient-centered care in three district hospitals in the Central Region of Ghana.

**Methods:**

The study was exploratory research that used qualitative methods to collect data from seven senior managers and 3 junior managers in three district hospitals in the Central Region of Ghana. Data were collected with the aid of an interview guide and a checklist. Data were analyzed using content analysis.

**Results:**

Two main Organizational-level factors were identified, namely, facilitators and barriers of patient-centered care. Facilitators to patient-centered care included: 1) Leadership commitment. 2) Leadership support. 3) Training and education for patient-centered care. Patient-centered care barriers identified in the hospitals were: 1) Leadership conceptualization of patient-centered care. 2) Lack of goals and sufficient activities for patient-centered care. 3) Communication related challenges.4) Ownership type. 5) Degree of centralization. 6) Financial constraints.

**Conclusion:**

Organizational-level factors that promoted patient-centered care were fairly present in the hospitals. Yet, several other factors negatively affected patient-centered care in the hospitals. A suitable patient-focused intervention is recommended for implementation at the health system and institutional-levels to improve patient-centered care. Hospitals managers should develop suitable goals and activities to stimulate patient-centered care with the full participation of hospital employees and patients and families.

## Background

Patient-centered care (PCC) was introduced in the 1970s [1], but gained impetus after the Institute of Medicine included it as a dimension of quality of healthcare [2]. PCC has moved to the center stage on issues of healthcare delivery and has received enormous attention as an effective approach to delivering quality healthcare that improve patient experience of care [3–5].

PCC is defined as healthcare that establishes partnership among practitioners, patients, and their families (when appropriate) to ensure that decisions respect patients’ wants, needs, and preferences and that patients have the education and support they need to make decisions and participate in their own care [2]. Epstein & Street [6], also defined PCC as care that is relationship-based and makes the patient feel known, respected, involved, engaged, and acknowledged. The two definitions above point to the need to make patients the locus of service provision and highlight the significance of partnership and collaboration with patients and their families in healthcare delivery. However, Baker’s definition [2] brings to the fore the importance of patients’ education and support to the promotion of patient-provider dyadic relationship, which is crucial in ensuring that patients participate in decision making regarding their own care. In this study, we assess organizational-level factors that facilitate or impede PCC in three district hospitals in the central region of Ghana.

In spite of the growing knowledge on PCC in general, and efforts by the Ministry of Health (MOH) and the Ghana Health Service (GHS) to improve patient-centeredness and patient experience of care [5, 7], patient experience research and anecdotal evidence in Ghana report negative patient experience such as short time spent with patients, disregard for patients’ questions, unauthorized collection of money from patients, disrespect and discrimination and poor education of patients [8–10]. To address these challenges, the GHS and other agencies of the MOH that provide healthcare services in Ghana are paying great attention to issues of service excellence, and have put in place patient centered strategies to improve patient experience of care [5–7]. In most healthcare institutions in Ghana, statements/phrases bordering on patient centeredness, client centeredness and client focus are often boldly displayed in hospitals as part of their mission and value statements.

This practice gives the impression that patient centeredness is the culture of hospitals in Ghana and that it is a shared principle and standard for providing healthcare in hospitals. However, there seem to be little empirical evidence on the level of preparedness and commitment of hospital leaders to the patient-centered values they espouse. Research on institutional-level facilitators and barriers to the achievement of the patient-centered vision of the health system and the patient-centered mission and value statements of healthcare institutions in Ghana appears to be missing. To fill these gaps in the healthcare quality literature on Ghana, this study aims at assessing organizational-level factors that enhance or inhibit PCC in three district hospitals in the Central Region of Ghana.

### Conceptual framework

This study is informed by the work of Dale Shaller [11]. Shaller in his work *Patient-centered care: what does it take?,* established seven key factors that contribute to PCC at the organizational level. The factors included 1) Leadership, 2) Involvement of patient and family at different levels, 3) Communication of the organization’s PCC strategic vision to members of the organization, 4) Creating supportive work environment for care givers, 5) systematic measurement and feedback, 6) Supportive technology and 7) Quality of the built environment. Leadership is an important factor of PCC [11]. It sets the direction for organizations by developing and managing its culture, delivering services and ensuring effective governance [11–16]. Empirical evidence emphasizes leadership commitment and support in terms of setting the stage, providing direction and resources for PCC [15–18]. Commitment of leadership to PCC determines the extent to which healthcare organizations orchestrate a fit between PCC vision and strategic plan and priorities as well as daily operational activities of health institutions [11]. Leadership has the responsibility not only to create a clear vision for PCC but also communicate and disseminate PCC philosophy and values among staff, integrating PCC values and activities into human resource policies and the day-to-day routines of all units and departments [17, 19, 20]. For instance, it has been found that articulating the organization’s patient-centered mission during staff orientation promote PCC [17].

Appropriate power sharing between providers and patients is one core attribute of PCC [21] and is related to patient and family involvement in care decision making. Patient and family can be involved in healthcare decision making at different levels of the organization. Studies have mentioned the setting up of patients’ advisory committee and involving them in organizational decisions such as service design, recruitment interviews and quality improvements activities as the means by which healthcare organizations can involve patients and family in decision making regarding their health [17]. Furthermore, care for providers and provision of quality work environment are essential organizational-level factors that promote PCC [22]. Work environment has several dimensions including social support, suitable working conditions, job characteristics, staff training and development and communication [22–24]. These form essential component of staff motivation and has implications for health professionals’ performance and quality patient experience of care [17, 25, 26].

Measurement of PCC related performance and gathering of patients’ feedback are central to continuous quality improvement in healthcare [11]. Patient feedback may include collecting data on patients’ care experience through surveys, direct observation, mystery shopping and use of patient complaint databases [17, 27]. To support systematic measurement and continuity of care, it is suggested that healthcare organizations should invest in technology such as health IT systems [11, 27]. For example, it has been argued that patient-focused information systems have the potential to promote access to patient and family data, encourage patient participation in healthcare and economize office space [27, 28]. It is therefore expected that leadership of healthcare organizations will invest in health IT to support PCC. According to Shaller [11], the environment in which care is delivered is very critical to PCC. The physical environment where care is delivered is said to be closely linked with the healing process and patient experience of care [11]. For example, studies have found that patients’ evaluation of quality of care are mostly based on physical cues such as quietness and cleanliness of the physical environment and physical space, health facility and colors [29, 30]. This makes the built environment an important factor of PCC. Thus, the above organizational-level factors can be viewed as “catalytic agents” of PCC.

**Fig. 1:**
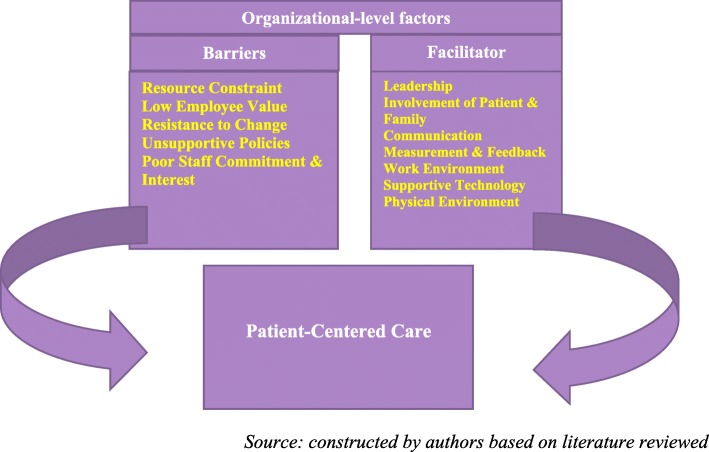
Facilitators and Barriers of PCC. *Source: constructed by authors based on literature reviewed.*

Even though Shaller’s work did not consider barriers to PCC, literature has detailed resource constraints, resistance to change, low employee value, unsupportive policies, and poor staff commitment and interest as organizational-level factors inimical to PCC [14, 17, 23, 26]. For instance, human, time and financial resource constraints have been mentioned as challenges to the delivery of PCC, particularly, limited staff capacity, insufficient staff, limited time to set the stage for PCC, limited office space and lack of equipment [17, 18]. In other studies, difficulties related to cultural change implementation, inexperienced staff, high workload and poor attitude of staff, stickiness to traditional or standard hospital practices and low employee value have been acknowledged to hamper PCC [14, 16, 17, 21, 28]. These factors can be viewed as frictional forces at the organizational-level that inhibit PCC effort. Figure 1 explains how facilitating and inhibiting factors at the organizational-level affect PCC. From Fig. 1, organizational-level barriers and facilitators of PCC exists side-by-side. The arrow that links the facilitators to PCC moves in a clockwise direction indicating the catalytic ability of the facilitators to propel PCC in the “right” direction. On the other hand, the arrow that links the barriers to PCC is anticlockwise in direction, emphasizing the inhibiting power of the barriers to hamper PCC efforts from becoming successful. Healthcare organizations can improve PCC by strengthening the facilitators of PCC and reduce the inhibitors or barriers to PCC.

## Methods

### Study design

The study is an exploratory research that used qualitative research design and in-depth interviews to collected data.

### Sampling

The study population included all district hospitals in the Central Region of Ghana and all hospital management members. Sampling was done in 3 stages. The first stage involved convenience sampling of 3 districts out of the 20 districts in the Central Region on the basis of proximity and lower cost. At the second stage, 3 district hospitals were purposively selected out of health facilities in the 3 districts for inclusion in the study based on size (30+ bed) and appropriateness of management and administrative systems (as specified in Act 525 of 1996) [31]. The third and final stage of the sampling involved purposive selection of study participants. Ten hospital staff were selected for participation in the study on the basis that their position in the hospitals made them better placed to respond to the interview questions. Participants included 4 staff from St. Luke Hospital, 3 staff from Effutu Municipal Hospital and 3 staff from Ejumako District hospital. In all, 3 Hospital Administrators, 3 Nurse Managers, 3 Training Coordinators and 1 Human Resource Manager participated in the interviews. The nurse managers and hospital administrators were members of the core management team of the hospitals in the study. The remaining participants were members of the hospitals’ management committee. To be included as a participant, a staff must have worked for 2 or more years, must be at least a management committee member and a permanent staff of the hospital. Table 1 presents demographic characteristics of the respondents.

**Table 1:** Demographic Characteristics of Respondents

Description	Freq.	(%)	Description	Freq.	(%)
*Age*			*Religion*		
*31–40*	6	60	Christian	10	100
*41–50*	3	30	Muslim	0	0
*50+*	1	10	Others	0	0
*Gender*			*Years Served in present position*		
Male	4	40	1–5	7	70
Female	6	60	6–10	2	20
*Education*			10+	1	10
Tertiary	10	100			
*Position of Respondents*					
Hospital Administrators	3	30			
Nurse Managers	3	30			
Human Resource Managers	1	10			
Training Coordinators	3	30			

*Source: Constructed by authors from field data*

### Study institutions

Healthcare in Ghana is organized in three levels (i.e., primary, secondary and tertiary). District hospitals are primary health facilities and serve as first-point of referral at the primary level. The Central Region of Ghana has 20 districts and each district has a district hospital. Two Ghana Health Service hospitals (Effutu Municipal Hospital and Ajumako District Hospital) and one private not-for-profit provider (St. Luke Catholic Hospital) participated in the study. These hospitals were selected from the Effutu Municipal, Gomoa Westand and Ajumako-Anyan-Assiam districts respectively. The three districts have an estimated population of 79,411, 153,570 and 160,813respectively. Healthcare is organized at the district, sub-district and community levels in each of the districts. Predominant economic activities in the first two districts are commerce, farming, fishing, fish mongering and salt winning. On the other hand, the economy of Ajumako-Anyan-Assiam District is predominantly farming. Effutu Municipal Hospital has143 bed capacity and 344 different categories of health staff. St. Luke Catholic Hospital is a 103 bed hospital with 235 staff strength and Ajumako District Hospital is a 55 bed hospital with 184 staff strength. Each of the three hospitals has a core management team made up of a Medical Superintendent, a Health Service Administrator, a Nurse Administrator and other Key Functional Heads. Other important teams and committees of the hospitals include the management committee, quality improvement teams and quality assurance committees.

### Data collection

In-depth interviews were conducted to collect data with the aid of an interview guide and a checklist. Both instruments were developed based on extensive literature review on organizational-level factors related to PCC [11, 13, 16, 17, 32]. Checklist and interview guide were checked for content validity. Data collection instruments were given to experts to validate their appropriateness in achieving the objectives of the research. Written permission and informed consent was obtained from management of the three hospitals. Appointments were scheduled with management of the selected hospitals for a face-to-face interview. Respondents were briefed about the purpose of the study and written informed consent was given by individual participants before each interview. The check list was used in verifying the existence or availability of strategic plans, training manuals, job descriptions, mission and vision statements, appraisal reports, client satisfaction survey reports, among others. Interviews were conducted by the first author on different days for each of the three hospitals, and recorded by a research assistant using field notes and an audio recorder. To reach saturation, authors requested to interview other relevant functionaries of the hospitals to gain more insight into barriers and facilitators of PCC in the hospitals. Authors also ensured that discussions with informants continued until no new barriers and facilitators of PCC were identified after interviewing participants in each of the hospital. Interviews lasted between 40 and 60 min. All interviews were in English language. Data were collected in February, 2019.

### Data management and analysis

Content analysis was used in analyzing data for the study. The audio data were transcribed immediately after the close of interview with each hospital by a research assistant. Field notes were also typed and properly organized. To ensure validity of the study results, responses validation and reflexivity were applied during data collection and analysis. Transcribed audio data and typed field notes were compared to identify omissions and were finally merged into a single transcript. The first author compared the transcribed data with the audio tapes to ensure that data on the tapes were captured accurately in the text. The text was put into a two column table format (first column for text and second column for making notes). The script was read through many times by both authors and brief notes on important information were made in the second column. The position of the first author as a former Health Service Administrator was reflected upon during data collection and analysis to ensure that her experience and knowledge of some contextual factors do not unduly influence interview discussions and data analysis. Both authors thoroughly read through the initial notes to identify and document relevant information which were grouped into categories. The categories were compared with the initial notes extracted from the data. These were then discussed and revised by authors and were grouped into categories and sub-categories and were further compared to the typed script by the second author to ensure that relevant information were not left out.

## Results

Two main categories of organizational-level factors of PCC were established, namely, facilitators and barriers of PCC. Patient-centered care facilitators had three sub-categories including leadership commitment, leadership support and training and education for PCC. Six sub-categories of PCC barriers were also established, comprising, leadership conceptualization of PCC, lack of goals and sufficient activities for PCC, Communication related challenges, ownership type, Degree of centralization and Financial constraints. The details are provided in Table 2.

**Table 2:** Organizational-level Factors of PCC

Main Categories	Sub-Categories
PCC Facilitators	Leadership commitment
	Leadership support
	Training and education for PCC
PCC Barriers	Leadership conceptualization of PCC Lack of Goals and Sufficient Activities for PCC Communication related challenges
	Ownership type
	Degree of centralization
	Financial constraints

*Source: Constructed by authors from field data*

### Patient-centered care facilitators

Three sub-categories of facilitators of PCC emerged in the study. They were, leadership commitment, leadership support and training and education for PCC.

### Leadership commitment

Interviewees from all three hospitals indicated commitment on the part of hospital management for PCC. Hospitals had “quality healthcare” and “patient-centeredness” boldly displayed by their mission and value statements indicating their commitment to and passion for delivering patient-centered care. The following are excerpts from the mission and value statements of the hospitals.

*Providing............high quality healthcare which is client centered……..,through highly motivated and trained staff.* (Excerpts of mission statement of hospital 1).

*.........committed to a client centered, quality driven, result oriented health care.......,through appropriate training in new skills....... and a highly motivated staff* (Excerpts from mission statement of hospital 2).

*The patient is number one; we respect the dignity of patients; we are patient-centered.* (Excerpts from value statements of the hospitals).

Other management actions that were identified to be supportive of PCC were the formation of committees that work to identify quality gaps and develop interventions to improve quality of healthcare. In two of the hospitals, it was mentioned that the quality assurance committee plan and carry out specific and general quality improvement interventions in the hospitals and have focal persons at various units responsible for implementation and coordination of improvement initiatives.

*We have Quality Assurance and Quality Improvement committees charged with the responsibility to ensure that healthcare provided to patients are of good quality.* (Administrator with 19 years practice experience).

The interviewees emphasized hospital management responsiveness to patients’ feedback and well-being. Two respondents from one of the hospitals referred to the inclusion of patients’ needs and feedback in the agenda of core management meetings. The respondents mentioned that hospital management involves unit heads in such meetings to outline solutions to patients’ concerns. Such practices they claimed have enhanced PCC in the hospital. The respondents cited continuous dissemination of management decisions meant to improve quality of care among staff and consistent monitoring of the implementation of such decisions as efforts on the part of the hospital’s management that sustains PCC in the hospitals.

*Patients’ complaints and feedback are discussed at management meetings. We involve unit heads in such meetings to provide solutions to patients’ concerns. Where necessary, we hold emergency staff durbar to disseminate among staff management decisions aimed at improving patients’ care.* (Administrator with 5 years practice experience).

### Management support

In all three hospitals, management support for PCC was mostly related to provision of logistics for healthcare delivery, staff training and incentives and continuous effort to improve the built environment of the hospitals. It was found in one of the hospitals that staff needs were identified and met through periodic staff satisfaction surveys. Other forms of management support found to facilitate PCC were provision of safe working environment and social support for hospital staff, as well as PCC role modeling by hospital leaders to inspire good attitude towards work and patients.

*.......our hospital conducts staff satisfaction surveys. We assess our performance from the perspective of staff. Through this, staff complaints about inadequate logistics and lack of motivation have received much attention. This motivate our staff to give their best to patients* (administrator with 5 years practice experience).

*Anytime I visit the wards, the manner in which I relate to staff and patients are commended by staff..........., the Nurse Manager does same............., the Medical Superintendent is accessible to pregnant women. He gives quality time to pregnant women during consulting, and responds promptly when called to attend to women in labor. We do this to inspire staff to build good interpersonal relationship with patients and relatives.* (Administrator with 7 years practice experience).

Respondents recounted management support for community durbars, community engagements, outreaches, patient satisfaction surveys and providing suggestion boxes at appropriate locations in the hospitals to solicit for feedback on its operations and services. Interviewees indicated that community engagements were done with the sole purpose of interacting with community members and patients to understand their needs and partner with communities to provide services that meet their needs. They mentioned that through community interactions and patient satisfaction surveys, the hospitals have been able to improve waiting time, staff attitude and provider-patient interaction.

*Waiting time was among the concerns raised by patients in the satisfaction survey. Based on the concerns a new consulting arrangement is in place. The medical assistant begins consulting early in the morning and the medical officers join later after ward rounds. The waiting time, I must say has reduced*. (Administrator with 19 years practice experience).

### Education and training for PCC

Periodic orientation of newly employed staff emerged as an important avenue for disseminating mission and value statements among staff. Interviewees indicated that hospital leaders take turns to share the mission and value statements during staff orientation to ensure that new staff understand and appreciate the patient-centered values of the hospitals. Periodic training on customer care for staff, monthly unit meetings, ward rounds and staff durbars were cited as platforms where issues related to patient care were discussed and shared to improve PCC. Problem-based learning was also identified as a facilitator to PCC. One of the participant indicated that through problem-based learning, the staff, in collaboration with patients and families have come up with initiatives to reduce cost of medication and improved care in their pediatric ward. However, none of the hospitals had patient representative committee in place.

*As a hospital we stand for our core values,........, during orientation, the administrator takes staff through our mission statement and core values to help staff understand what we stand for and to influence their behavior* (Training Coordinator with 3 years practice experience).

*Issue on patient care come up during ward rounds and ward meetings, we discuss and come up with small-scale interventions to improve on the quality of care for patients. We have one of such initiatives at the pediatric ward which has reduced cost of medication and improved care for children* (Nurse Manager with 3 years practice experience).

Other activities described to facilitate PCC included patient education, patient orientation, patient counseling and follow-ups. We found that these were avenues used by the hospitals to interact with patients and empower them to make informed decisions and choice regarding their health. Two of the respondents explained that patient counselling and the effective system of patient follow-up in their hospital has contributed to PCC. According to these respondents, their hospital has been able to move beyond meeting medical needs of patients to providing care that meet the physical, emotional and spiritual needs of patients through patient counseling and follow-up.

### Barriers to PCC

The study result identified six elements present at the health facilities that were unfavorable to PCC. They were leadership conceptualization of PCC, lack of goals and sufficient activities for PCC, communication related challenges, ownership type, degree of centralization and financial constraints.

### Leadership conceptualization of PCC

Respondents conceptualized PCC in various ways. Some respondents conceptualized PCC as all the things that go into patients’ care, i.e., provision of logistics, infrastructure, salaries, and training among others. Other respondents emphasized the development of nursing care plan for individual patients as PCC while others also viewed PCC as customer care which they defined to mean paying attention to patients and their needs and providing quality care for patients.

*........nursing care plan is about patients’ needs, so each patient has a nursing care plan that focus on the needs of the patient. The nursing care plan is about PCC.* (Nurse Manager with 2 years practice experience).

Patient-centered care was also conceptualized as putting measures in place to promote good staff attitude and respect for patients, maintaining clean hospital environment and ensuring supply of human and material resources for healthcare delivery.

*We believe good staff attitude is key to PCC...., we stress on attitude towards patients because a nurse can appear neat and well dressed, but if he/she does not treat patients well, what is his/her use.* (Nurse Manager with 3 years practice experience).

*If I request for more midwives, ensure supply of pharmaceuticals and a standby ambulance, it is to promote care for patients and it can be considered as PCC...., it is about everything we do here. We do it unconsciously.* (Administrator with 7 years practice experience).

### Lack of goals and sufficient activities for PCC

No specific PCC strategic goals or targets and guidelines were identified in the hospitals to express the exact improvement hospital managers intend to record on PCC. Also, job descriptions of the various professional groups didn’t seem to have specific responsibilities on PCC for staff. Further, an inconsistency was found between the mission and value statements and the patient-focus policy implemented by the hospitals. Whiles the mission and value statements pointed to PCC, the patient-focus policy in force was customer care.

### Communication related challenges

Respondents referred to language differences, fear of victimization, unwillingness of patients and families to report unfair treatment, ask questions or seek clarifications as impediments to patient education and efforts to involve patients in decisions related to care. This situation was said to affect patient-provider relationship, particularly nurse-patient relationship. One of the respondents specified that nurse-patient misunderstanding in her hospital was more of pseudo-conflict arising from supposed differences between nurses and patients, often resulting in stereotyping of nurses. Demand for unauthorized fees by caregivers was also cited as one of the reason for misunderstanding between caregivers and patients.

*Sometimes the perception of patients about nurses and language barriers result in misunderstanding between patients and nurses. A patient would come across a nurse and would think that because the nurse is neatly dressed, he/she is not likely to assist if the patient should ask for help.* (Nurse Manager with 2 years practice experience).

*...........nurse-patient misunderstanding that comes up at management meetings for discussion often relates to unauthorized payments demanded by staff* (Hospital Administrator with 19-years practice experience).

### Ownership type

Participants mentioned that ownership status of hospitals determine the policies and processes employed in managing hospitals, which also has immense implications for PCC. Three of the respondents referred to aspects of human resource, procurement and revenue management policies and procedures as having adverse effect on PCC. Although there were similarities in the policies, processes and procedures of the three hospitals, policies and procedures related to human resource management, procurement and revenue management of the mission-based hospital were more supportive of employee behavior management, reward and accountability and prompt supply of logistics when compared with those of the other two public hospitals.

*There are 54 medicines that cannot be sourced from the open market when they are not available at the regional medical stores. What happens if a doctor needs some of those medicines to work with and we cannot get it from medical stores? This is a policy from the top. They sit up there and conclude that private hospitals are performing better than us.........., we are unable to supply medicines on a timely basis because the regional medicals store does not have either* (Administrator with 19 years practice experience).

*Yes, patients are number one, but our staff are also number one. We operate a flexible policy. We have the mandate to provide monetary incentives to all staff and demand accountability from staff. As managers of the hospital, we can dismiss a staff if we deem fit.* (Administrator with 5 years practice experience).

### Degree of centralization

Ownership type had implications for the extent to which certain decision making authority is centralized or decentralized in the hospitals. Some managers expressed displeasure with the institutional arrangements in their hospitals and indicated that operational decisions and management actions were expected to remain strictly within the policy framework of their mother organization. Two of the respondents specified having limited authority for decisions related to hiring and dismissing of health professionals and provision of incentives. They stressed that the institutional arrangement under which they operate deny them opportunity to participate in the recruitment of employees they going to manage, thereby limiting their authority to demand accountability from staff. We identified that the centralized nature of such decisions accounted for non-centered behavior of some staff in the hospitals.

*We do not develop the job description of staff. All the job descriptions are provided by the Ghana Health Service and we cannot extensively vary them.* (HR Manager with 7 years practice experience).

*We work within the policies of our mother organization, so we cannot have a vision and mission entirely different from that of the parent body. We have to work within the policies and procedures set for us.*

*For poor staff attitude, hmm! You see, the disciplinary issues are difficult. Staff know we cannot sack them. From my experience, a misbehaving staff can report you to the head office.*

(Administrator with 19 years practice experience).

### Financial constraints

Interviewees indicated that inadequate funds holds back effort to provide optimum support in terms of good working conditions, technology and other key logistics for the delivery of quality healthcare. Respondents attributed delays in and inadequate supply of logistics and equipment, inability to implement adequate PCC interventions and poor staff incentives to financial constraints of the hospitals.

*Our staff wants monetary incentives, but we are able to provide general incentives such rice and oil at the end of the year and sometimes staff uniforms. This is due to financial difficulties.* (Nurse Manager 2 years practice experience).

*Due to lack of funds we have a challenge ensuring constant supply of medical equipment, non-medicine consumables and the required number of essential training programs for staff* (Administrator with 7 years practice experience).

## Discussion

This study assessed organization-level facilitators and barriers to PCC in three district hospitals in the central region of Ghana. We found that all hospitals in the study had mission statement and core values which identified them as patient-centered facilities. The patient-centered values and mission statements demonstrated leadership commitment to PCC. Such organizational statements are guide to achieving organizational vision and can signal to hospital employees that the hospitals are patient-centered facilities and that PCC is the standard principles that guide healthcare delivery in the hospitals. The results point to hospital managers’ interest in responding to patients’ feedback and discussing issues of the bedside in management meetings. This demonstrate some level of commitment on the part of management to PCC and is consistent with literature from United States of America (USA) where leaders of healthcare institutions with track records for improving patient experience devoted quality time to discuss issues of patient experience at meetings [17]. We also identified leadership support for PCC in terms of funding for staff training, provision of needed resources, community engagement and patient follow-up, which are essential in fostering the needed environment for available and responsive care [27]. Management support for PCC in this study corroborates findings from the USA [25].

It must be noted however that, the mission statement and core values of the hospitals were not supported by specific PCC strategic goals and activities. Having mission and value statements on PCC are not enough to provide PCC. It is important for the hospitals to outline the set of activities they intend to commit to in order to achieve their mission of patient centeredness. Further, the patient-focus policy in force at the hospitals was customer care. Customer care may suffice for improving patient experience of care or patient satisfaction with care but may not be sufficient to promote provider-patient collaboration and power sharing which is at the core of PCC [33]. The nonexistence of strategic goals, guidelines and activities to support the patient-centered values of the hospitals support findings from Ethiopia and the USA [34, 35].

The results indicated that managers conceptualized PCC differently from the constructs described in the PCC literature. To some extent it shows that hospital managers are likely to pursue initiatives that are not consistent with PCC. Inappropriate conceptualization of PCC can be a primary barrier to PCC because the manner in which hospital leaders conceptualize PCC inform their choice of initiatives and how those initiatives are developed and applied to improve PCC [36]. It is not surprising that all the hospitals implemented customer care instead of PCC. Improving patient experience of care is one of the strategic directions of the health system of Ghana [5, 7]. As such, it is vital that proven interventions for improving patient experience of care such as PCC is acknowledged and properly conceptualized by hospital managers. The lack of agreement between PCC conceptualization among hospital managers and PCC construct prescribed in the literature corroborate study findings from the USA and Ghana [36, 37].

The patient-centered value statement of hospitals makes PCC role modeling, reward and accountability important elements for shaping PCC culture in the hospitals. We found PCC role modeling on the part of managers as essential in promoting patient-centered behavior among staff. Continuous PCC role modeling will indicate to individual staff behaviors that are acceptable and those that are unacceptable in the hospitals [38]. However, the degree of centralization of decision making related to recruitment or dismissal of staff and staff incentives has the potential to weaken accountability. Decentralizing recruitment decisions to hospital management will allow managers to consider value similarities during recruitment. Similarly, employees will conform to prescribed behaviors if they believe their behavior would be rewarded or punished. Moreover, the problem of limited funds does not only affect hospital managers’ ability to reward PCC behavior of staff, it also affects the supply of resources required for the delivery of quality healthcare. Financial challenges related to supply of resources and provision of incentives to staff mirrors findings of previous studies [39, 40].

Good provider-patient communication is imperative to PCC. Therefore, communication related challenges identified in the study can affect therapeutic alliances between patients and providers if serious attention is not paid to it. Nurses spend lots of time with patients and are responsible for planning and administration of treatment to patients. In this regard, effective communication skills are indispensable to the discharge of their duties if hospitals can improve nurse-patient interaction and patient experience of care. Nurse-patient misunderstanding in this study confirms findings on nurses’ communication challenges with patients and family [41, 42].

### Limitations

The study was limited to 3 district hospitals in the central region of Ghana. The study considered organizational-level factors related to PCC only. Facilitators and barriers of PCC were considered from the perspective of hospital management and do not in any way reflect the views of hospital employees and patients. Also, nurse-patient communication was not considered in the study. Therefore, the findings do not represent views of nurses and patients on communication related challenges in the hospitals and must not be interpreted as such. A full investigation on provider-patient communication will be necessary.

## Conclusion

The study assessed organizational-level facilitators and barrier of PCC. Facilitators and barriers of PCC were both present at the organizational-level of the hospitals. Facilitating factors that propelled PCC in the right direction such as education and training for PCC, leadership support and commitment were fairly present in the hospitals. Yet, there were inconsistencies between patient-focus policies of the hospitals and their statement of commitment to PCC. The study identified barriers that hampered PCC effort of the hospitals, comprising lack of congruence in managers’ conceptualization of PCC and the constructs of PCC as prescribed in the literature. There were also interrelated factors such as ownership type, degree of centralization and financial constraints that inhibited efforts to promote PCC in the hospitals. Since the strategic vision of the health system is to promote patient centeredness and improve patient experience of care, it is important that the appropriate patient-focused intervention (i.e., PCC) is identified and implemented both at the institutional and system levels to achieve the patient-centered vision of the health system. PCC should be clearly conceptualized and defined by the health system leadership and hospital managers to guide formulation of PCC improvement initiatives. The GHS should grant hospital managers the measure of autonomy and flexibility necessary to strengthen staff commitment to PCC. Hospital managers should agree on strategic objectives and activities for improving PCC with the full involvement of hospital employees. Managers should as well consider giving patients a representation on their quality improvement and assurance committees for discussions and planning for PCC improvement initiatives.

## Supplementary information

**Additional file 1.** Title of data: interview guide. Description of data: the data collection instrument consists of the interview guide used in collecting data for the study.

## Data Availability

The datasets used and/or analyzed during the current study are available from the corresponding author on reasonable request.

## Abbreviations

GHS: Ghana Health Service
MOH: Ministry of Health
PCC: Patient-centered Care
