# Supplementary material for: Facilitators and barriers of patient-centered care at the organizational-level: a study of three district hospitals in the central region of Ghana
Source: BMC Health Serv Res. 2019 Nov 27;19:900. doi: 10.1186/s12913-019-4748-z (PMC6882059; doi:10.1186/s12913-019-4748-z)
Supplement: Supplementary file 1 — Additional file 1. Title of data: interview guide. Description of data: the data collection instrument consists of the interview guide used in collecting data for the study. [file 12913_2019_4748_MOESM1_ESM.docx]

**Appendix I**

**INTERVIEW GUIDE**

**Preamble**

This instrument seeks to assess factors that enhances patient-centered care (PCC) in hospitals in the Central Region of Ghana. Data collected in this research will be used purely for academic work /publications. Anonymity of all respondents in this study is guaranteed. We would therefore be grateful if management and relevant staff of this hospital could grant us interviews on the following issues related to PCC in this hospital.

**Part One**

1. How do you understand PCC?
2. What are the vision, mission and value statements of this hospital and how do they support PCC?
3. Do you have policies on PCC?
4. What initiatives are available for promoting and sustaining PCC in this hospital?
5. What has been the role of management in promoting PCC in this hospital
6. How do ensure that staff share in the mission and value statements of the hospital?
7. Do you have a strategic plan? If yes, what are some of the strategic goals on PCC?

**Part Two**

1. Which of the activities mentioned below do you employ to ensure that employees share in your mission and values statements and how do they promote PCC?
2. Display of vision and mission statement of the hospitals at vantage points in the hospital
3. Circulation of flyers and brochures to patients and staff
4. Staff orientation and training
5. PCC role modeling by hospital leaders
6. Periodic meetings to discuss PCC
7. HR handbooks, policies and procedures
8. Staff recruitment interviews
9. Sharing patients’ stories at staff meetings

**Part Three**

1. Do you have the following activities in place? How do you carry them out and how do they support and promote PCC in this hospital?
2. Developing activity plans for PCC
3. Integrating PCC into job description of hospital employees
4. Setting PCC performance objectives with staff
5. Providing supportive work environment for staff
6. Ensuring constant supply of logistics for service delivery
7. Evaluation of patient satisfaction/experience of care
8. Patient education and interaction
9. Incorporating patients and staff views into PCC initiatives
10. Reward and accountability system for improving PCC

**Part Four**

1. Which of the following structures are in place for enhancing PCC and how do they promote PCC in this hospital?
2. Patients’ advisory committee
3. PCC teams
4. PCC focal persons
5. Clients’/patients’ information desk
6. Suggestion boxes for patients and staff
7. What are the challenges to the implementation and promotion of PCC in this hospital?

**Part Five – Nursing Administration**

1. How do you understand PCC?
2. How are patients and their families involved in care decisions?
3. Are issues of patient care and patients’ feedback allowed for discussion at management meetings?
4. What are some of the issues and what has been the response of hospital management to those issues?
5. Do you agree on PCC goals with Nurse In-charges when setting objectives for performance appraisal? If yes, what are the specific objectives?
6. Do you provide specific orientation for nurse interns, nurses on transfer and newly appointed nurses?
7. What has been the content of the orientation and how does it promote PCC?
8. Do you hold meetings with nurses to discuss ways of providing patient-centered services to clients?
9. How often is this done and what are some of the things you discuss?
10. What challenges do the nursing administration face in its effort to promotion of PCC?
